# Introduction

**DOI:** 10.1093/eurpub/ckae114.fm001

**Published:** 2024-09-26

This supplement is published in conjunction with the 15th conference of HEPA Europe, the WHO Regional Office for Europe network for the promotion of Health-Enhancing Physical Activity.

The HEPA Europe 2024 conference will focus on ‘Optimising health enhancing physical activity: the importance of inclusion’. The objective of the conference is to engage and connect scientists, policymakers, professionals and other stakeholders to enhance and facilitate the implementation of knowledge in the field of Health-Enhancing Physical Activity. Bringing together leading experts, young researchers, policymakers, professionals and other stakeholders, the HEPA Europe 2024 conference in Dublin provides a unique forum to stay up-to-date with scientific, practice and policy developments in Europe and beyond.

The focus of this year’s conference is of particular importance as we know that levels of physical activity are lower in certain population groups e.g. women, older adults, and those with chronic disease and disability. Indeed, there has been a recent call that research efforts should focus on ‘*understudied areas and underserved populations, and use new methods for implementing and scaling up physical activity interventions’*, in a common mission for equitable improvement of population health and wellbeing through active living.^1^ We need to ensure that programmes and opportunities to be active are appropriately tailored for these populations groups, and that we can remove any barriers to participation in health enhancing physical activity.

At this year’s conference we will hear from three experts in the field who will explore our conference theme from a variety of perspectives. Dr Chris Gidlow’s talk will consider inequities observed in various contexts in “Thinking about inclusion - learning from physical activity research and beyond”. Dr. Jane Thornton will share insights on “A call for movement equity: What (and who) are we still missing in the conversation on physical activity and health?”,^2^ and Professor Adrian Bauman will be delving into: “Systems approaches to physical activity – becoming commonplace or still a rare occurrence?”.

We also have 16 symposia and 220 oral or pitch presentations from research, practice and policy perspectives. This will provide a rich opportunity for engagement, supporting a move away from silos and minimising well established disconnects between academic research, policy and practice.^1^ Oral and pitch presentations are grouped under 20 themes ranging from chronic conditions to paediatric disability to nature-based interventions and mental health. This diversity of themes shows the breadth of expertise among researchers, practitioners and policy-makers in health-enhancing physical activity. We encourage delegates to attend not just sessions in your own areas of expertise, but to broaden your horizons by immersing in a less familiar theme. We hope this will lead to cross-fertilisation of ideas between sectors and the opportunity to be creative in learning from others.
